# Weaponizing the Appraisal: Justice Violations, Psychological Safety, and Employee Voice in a Saudi Multinational Organization

**DOI:** 10.5334/pb.1519

**Published:** 2026-09-23

**Authors:** Mohamed Mohiya

**Affiliations:** 1 College of Business, Human Resources Management Department, King Khalid University, Abha, Saudi Arabia

**Keywords:** Organizational Justice, Psychological Safety, Employee Voice, Human Resource Management, Workplace Dignity, Saudi Arabia, Reflexive Thematic Analysis, Single-Case Study

## Abstract

**Background:**

Conduct that degrades employees — belittling speech, the coercive use of rank, and treatment stratified by contractual or national category — ranks among the most consequential and least tractable problems confronting the human resource (HR) function, which authors the policies prohibiting such conduct, administers the channels through which it is reported, and answers for the outcome when a report yields nothing. This study asks how such conduct persists within an organization that formally forbids it, and where HR can intervene, by reconstructing the fairness appraisals and safety calculations that employees use to decide whether to speak. Evidence bearing on that question has accumulated overwhelmingly in Anglophone, individualist economies, leaving scholarship and practice alike thinly provisioned for hierarchical Gulf workplaces, where deference norms, kinship obligation, and stratified expatriate labor markets condition what employees regard as sayable.

**Purpose:**

This theory-driven exploratory study investigates three questions of a single large Saudi-headquartered multinational: how personnel interpret the unacceptable conduct they encounter or witness, why so few translate that interpretation into a formal complaint, and which organizational arrangements hold the resulting pattern in place.

**Theoretical Framework:**

Two established lenses are combined. Organizational Justice Theory (OJT; Colquitt, 2001; Greenberg, 1987) supplies the vocabulary through which employees appraise the fairness of outcomes, procedures, and interpersonal treatment. Psychological Safety Theory (PST; Edmondson, 1999; Kahn, 1990) provides the vocabulary for perceived risk associated with speaking. Read jointly, the two treat harmful conduct not as isolated incivility but as a fairness appraisal that simultaneously recalibrates the felt cost of voice.

**Methodology:**

Evidence was assembled within a single bounded case and drawn from two registers of employee talk. The first comprises 197 unsolicited postings left by staff on a company-wide internal communication platform. The second comprises thirty-nine semi-structured conversations held privately with employees across operational, professional, supervisory, and senior grades, and across permanent and agency contracts, spanning four nationality groupings. Both corpora were interpreted through reflexive thematic analysis (Braun & Clarke, 2006, 2019), double-coded on a stratified subsample, and read against one another for convergence.

**Key Findings:**

The analysis produced five categories. Supervisory authority was misused, most consequentially when appraisal ratings were used as leverage over compliance. Concerns were then withheld as a reasoned response to observed retaliation rather than as passive acceptance. Beneath both lay structural arrangements — contractual standing and personal connection — that distributed dignity before any encounter took place. Declared commitments to respect diverged visibly from enacted practice, and that divergence was itself read as evidence of where institutional protection lay. Cultural, religious, and generational registers supplied the terms in which employees judged conduct and justified their responses.

**Contributions:**

The study formalizes the Justice–Safety Vicious Cycle Model, a six-stage account in which each fairness violation lowers the perceived safety of speaking, each withheld concern removes the information that would otherwise trigger correction, and the resulting procedural dormancy licenses the next violation. Empirically, it supplies rare interior evidence from a Saudi industrial workplace and isolates the weaponization of performance appraisal as a distinct instrument of control. Methodologically, it demonstrates what is gained by reading spontaneous internal discourse alongside elicited interview accounts. For HR, the findings relocate the problem from individual supervisory temperament to the systems the function itself designs and administers — appraisal calibration and appeal, the separation of evaluation from investigation, enforceable protection for those who report, parity of treatment across employment categories, and merit-based personnel decisions — and the model identifies where in the cycle each of these levers takes effect.

## 1. Contextual Background and Research Rationale

### 1.1 Setting the Scene

The organizational imperative to construct ethically sound, respectful, and psychologically safe workplaces has received escalating attention from scholars and practitioners alike, given its deep implications for employee welfare, sustainable performance, and long-term institutional legitimacy (Leake et al., 2025; Porath & Pearson, 2013; Edmondson & Lei, 2014). The manner in which employees are treated within hierarchical structures profoundly shapes their emotional investment, sense of belonging, task engagement, and mental health (Mensah et al., 2026; Colquitt et al., 2013; Einarsen et al., 2020; Mohiya, 2026c). Conversely, when organizations tolerate or perpetuate mistreatment behavior — manifested through verbal hostility, coercive use of managerial power, discriminatory conduct, persistent bullying, or the systematic suppression of dissent — the consequences ramify across individuals, teams, and institutions (Hershcovis, 2011; Tepper et al., 2017).

Accumulating meta-analytic evidence has established the pervasive and damaging reach of workplace mistreatment. Employees exposed to abusive supervisory conduct, incivility, or discriminatory treatment report elevated rates of emotional depletion, psychological distress, compromised occupational satisfaction, weakened organizational commitment, and intensified withdrawal intentions (Leake et al., 2025; Mackey et al., 2021; Schilpzand et al., 2016; Liang et al., 2022). Beyond direct victims, witnessing colleagues being mistreated generates vicarious distress and corrodes the shared psychological safety necessary for team learning and collective effectiveness (Dollard et al., 2026; Treviño et al., 2014; Frazier et al., 2017). At the organizational level, such dynamics translate into measurable declines in productivity, inflated attrition costs, legal exposure, and reputational deterioration (Pearson & Porath, 2009).

Despite these well-documented consequences, scholarly understanding of how employees subjectively experience, interpret, and behaviorally respond to mistreatment behavior remains considerably incomplete, particularly when organizational settings depart from the Western individualist assumptions embedded in much existing research (Aycan, 2005; Pellegrini & Scandura, 2008). The Gulf Cooperation Council region, broadly, and Saudi Arabia specifically, constitute a critically underexamined terrain in organizational behavior scholarship (Mellahi & Budhwar, 2010; Budhwar & Mellahi, 2016).

### 1.2 Knowledge Gaps and Research Problem

Notwithstanding growing scholarly interest in ethical workplace conduct, multiple critical knowledge lacunae persist. First, quantitative survey methodologies predominate in this literature, yielding limited insight into employees’ phenomenological experiences, meaning-making processes, and contextually embedded responses to mistreatment (Lutgen-Sandvik, 2006; Tracy et al., 2006). Authentic qualitative accounts of lived experiences of mistreatment remain comparatively scarce.

Second, explicitly theory-grounded qualitative investigations that systematically situate employee accounts within established conceptual frameworks are notably absent; many qualitative studies adopt purely descriptive orientations that curtail theoretical development (Tracy et al., 2006). Third, the geographic skew toward Western organizational contexts severely limits the field’s cross-cultural generalizability (Cortina et al., 2017; Budhwar & Mellahi, 2016). Fourth, insufficient scholarly attention has been paid to the roles of organizational silence and psychological safety as possible mediating mechanisms linking mistreatment to its individual and collective consequences (Dollard et al., 2026; Morrison, 2014; Newman et al., 2017). Fifth, the intersection of employment status vulnerability with mistreatment exposure and justice perceptions remains inadequately theorized (Liden et al., 2003; Gallagher & McLean Parks, 2001).

### 1.3 Theoretical Underpinning

To address these gaps, this study adopts an integrated conceptual framework that draws on Organizational Justice Theory (OJT; Colquitt, 2001; Greenberg, 1987) and Psychological Safety Theory (PST; Edmondson, 1999; Kahn, 1990). This pairing is theoretically motivated by empirical evidence suggesting that both fairness appraisals and perceptions of interpersonal safety shape employees’ experiences of mistreatment.

Organizational Justice Theory offers a multi-dimensional architecture for appraising workplace treatment. Distributive justice concerns the equitability of outcome allocations (Adams, 1965); procedural justice addresses the fairness of decision-making processes (Leventhal, 1980; Thibaut & Walker, 1975); and interactional justice captures the quality of interpersonal dignity extended during organizational transactions (Bies & Moag, 1986; Colquitt, 2001). Mistreatment behavior constitutes a fundamental violation of interactional justice — conduct that strips individuals of dignity, courtesy, and informational transparency (Bies, 2015).

Psychological Safety Theory addresses individuals’ shared beliefs about the interpersonal risk of speaking up or taking action in organizational settings (Edmondson, 1999). Where psychological safety obtains, employees can voice concerns, flag errors, and challenge authority without anticipating punishment or marginalization (Kahn, 1990). Its erosion produces organizational silence — the collective suppression of potentially corrective speech — with profound consequences for learning, accountability, and institutional health (Morrison & Milliken, 2000).

The integration is theoretically synergistic: justice violations communicate to employees that environments prioritize protecting power over protecting people, directly degrading psychological safety (Nembhard & Edmondson, 2006). Simultaneously, eroded psychological safety prevents employees from voicing concerns that might disrupt ongoing justice violations, reinforcing unjust structures (Dollard et al., 2026; Detert & Burris, 2007). This bidirectionality generates self-sustaining cycles of mistreatment and silence that are resistant to superficial intervention.

### 1.4 Research Aim, Objectives, and Questions

This study aims to develop a rich, contextually situated, and theory-grounded understanding of how employees within a large Saudi multinational organization experience, interpret, and respond to mistreatment, with particular attention to the interplay among perceptions of organizational justice, psychological safety conditions, and employee voice behavior, and to the part played by the performance management system in shaping each.

The overarching aim generates a coherent hierarchy of inquiry. The five objectives operationalize the aim by directing attention to specific empirical and analytical tasks — characterizing behaviors, interpreting them through justice frameworks, examining safety dynamics, tracing mechanisms of silence, and analyzing contextual factors that shape employees’ experiences of and responses to such conduct. The five research questions then translate each objective into a concrete investigative focus, ensuring that every question can be traced directly to an objective and, through it, to the overarching aim. This nested structure reflects a deliberate analytic logic grounded in the study’s integrated theoretical framework.

*Research Objectives:*

- To identify and characterize the principal forms of mistreatment behavior encountered by employees in the research setting
- To examine how employees appraise mistreatment experiences through organizational justice frameworks
- To investigate the relationship between mistreatment exposure and psychological safety conditions
- To explore the mechanisms through which organizational silence is produced and maintained
- To analyze how contextual factors — cultural, structural, and positional — shape mistreatment experiences and responses

*Research Questions:*

- RQ1: What forms of mistreatment behavior are identified by employees as most consequential, and how are their impacts characterized?
- RQ2: How do employees’ mistreatment experiences intersect with their perceptions of organizational justice?
- RQ3: What is the nature of the relationship between mistreatment behavior, psychological safety, and voice behavior?
- RQ4: How do organizational systems, structures, and practices sustain or perpetuate mistreatment behavior?
- RQ5: In what ways do cultural values, hierarchical arrangements, and employment precarity shape how employees experience mistreatment and how they respond to it?

## 2. Conceptual and Empirical Foundations

### 2.1 Defining Mistreatment Behavior

Mistreatment behavior refers to harmful interpersonal conduct that violates organizational dignity norms, social civility standards, and foundational principles of human respect (Leake et al., 2025; Pearson et al., 2001; Hershcovis, 2011). The scholarly literature employs a constellation of overlapping constructs — workplace aggression (Neuman & Baron, 1998), incivility (Andersson & Pearson, 1999), abusive supervision (Tepper, 2000), bullying (Einarsen et al., 2020) — that share the defining feature of actual or potential harm to employees’ welfare, dignity, or professional standing.

This study adopts an inclusive working definition: mistreatment behavior encompasses any conduct that (1) transgresses organizational values affirming dignity and respect; (2) cultivates hostile, demeaning, or intimidating workplace climates; (3) exploits asymmetric power for personal gain or retaliatory purposes; (4) discriminates based on protected or categorical characteristics; or (5) systematically suppresses or punishes employee voice. This definition deliberately encompasses both high-intensity singular incidents (direct threats, public humiliation) and cumulative low-intensity patterns (persistent exclusion, dismissiveness) whose aggregated harm is equally corrosive. This definition is grounded in the empirical evidence reviewed in Section 2.5, which documents that the most harmful workplace behaviors empirically share these characteristics: they involve power exploitation (Tepper et al., 2017), violate dignity norms (Bies, 2015), suppress voice (Morrison, 2014), and generate cumulative harm irrespective of intent (Cortina et al., 2013).

### 2.2 Organizational Justice Theory: A Multi-Dimensional Framework

Organizational Justice Theory constitutes an integrative conceptual system for examining how employees evaluate the fairness of their organizational experiences and how these evaluations inform their attitudes, emotions, and behavioral responses (Colquitt, 2001; Greenberg, 1987; Cohen-Charash & Spector, 2001).

Distributive justice concerns whether the allocation of organizational outcomes — rewards, recognition, advancement, sanctions — conforms to applicable equity, equality, or need-based principles (Adams, 1965). Procedural justice evaluates the fairness of the processes through which organizational decisions are reached (Leventhal, 1980; Thibaut & Walker, 1975). Leventhal’s (1980) criteria stipulate that fair procedures must be applied consistently, free from bias, grounded in accurate information, correctable, representative of affected parties, and consistent with prevailing ethical standards. Interactional justice addresses the quality of interpersonal conduct experienced during organizational processes, comprising interpersonal justice (treatment with dignity, courtesy, and respect) and informational justice (provision of adequate and transparent explanations) (Bies & Moag, 1986; Colquitt, 2001). While analytically distinct, the three justice dimensions are empirically correlated and interact in practice: interactional injustice (e.g., verbal humiliation by a supervisor) typically co-occurs with procedural injustice (biased complaint processes) and distributive injustice (unequal allocation of sanctions), creating compounded fairness deficits whose combined harm exceeds any single dimension (Colquitt et al., 2001; Ambrose & Schminke, 2009).

Meta-analytic evidence consistently reveals that justice perceptions powerfully predict organizational commitment, institutional trust, task performance, withdrawal behavior, and psychological well-being (Colquitt et al., 2013; Cropanzano et al., 2017). The ‘target similarity principle’ (Rupp & Cropanzano, 2002) further specifies that interactional injustice is most consequential when supervisor-directed, while procedural injustice carries stronger implications for organization-directed outcomes. Recent scholarship continues to affirm the framework’s vitality: Colquitt and Zipay (2015) consolidated the construct’s conceptual and measurement architecture, while Howard et al. (2020) documented how experiences of workplace exclusion shape employees’ attitudes and behavior. Koopman et al. (2022) further confirmed the predictive reach of such perceptions for citizenship and contextual performance. Qualitative evidence from Saudi organizations, in the same vein, indicates that employees construct working theories of fairness from observed leader conduct rather than from formal policy statements (Mohiya, 2026h).

### 2.3 Psychological Safety Theory and Organizational Silence

Psychological safety, as originally conceptualized by Edmondson (1999, p. 350), refers to ‘a shared belief held by members of a team that the team is safe for interpersonal risk-taking.’ In psychologically safe environments, individuals engage authentically, raise concerns freely, admit errors openly, and challenge prevailing assumptions without anticipating reputational damage, social sanctioning, or career detriment (Kahn, 1990). Where such safety is absent, organizational silence prevails — employees systematically withhold observations, concerns, and critical information, depriving organizations of the upward feedback necessary for learning and correction (Morrison & Milliken, 2000).

Edmondson’s (2003, 2019) work identifies leader behavior as a primary determinant of psychological safety: leaders who model accessibility, explicitly invite dissent, acknowledge fallibility, and respond non-defensively create the conditions for voice. Conversely, punitive responses to challenge, public shaming, or coercive use of evaluation authority actively destroy psychological safety (Dollard et al., 2026; Frazier et al., 2017; Edmondson & Lei, 2014). Frazier et al.’s (2017) meta-analysis of 136 studies confirmed psychological safety as a robust predictor of organizational outcomes, while Edmondson and Bransby (2023) recently emphasized its increasing relevance in rapidly changing organizational environments. Evidence from hierarchical organizations converges on the same conclusion from the opposite direction: discretionary contribution is released only once employees judge that a threshold of perceived safety and organizational backing has been crossed (Mohiya, 2026e, 2026i).

Employee voice — ‘the discretionary communication of ideas, suggestions, concerns, or opinions about work-related issues with the intention of improving organizational or unit functioning’ (Morrison, 2011, p. 375) — represents a core behavioral expression of psychological safety. Research consistently identifies fear as the principal barrier to voice (Dollard et al., 2026; Morrison, 2014; Detert & Treviño, 2010): employees remain silent when they anticipate reputational harm, relationship damage, or material career consequences. These fears intensify dramatically when the subject of concern involves powerful organizational members (Milliken et al., 2003). Related evidence on knowledge sharing and hiding indicates that withholding is frequently a deliberate, calculated response to perceived organizational conditions rather than a symptom of disengagement (Pereira & Mohiya, 2021).

Morrison and Milliken (2000) distinguished analytically productive individual silence from systemic organizational silence, which emerges when broadly shared beliefs develop that voicing concerns is either futile or dangerous. This systemic condition generates cascading organizational pathologies: problem identification fails, cynicism spreads, accountability erodes, and mistreatment persists unchallenged (Vakola & Bouradas, 2005; Liang et al., 2022).

### 2.4 Bidirectional Justice–Safety Linkages

The conceptual linkage between justice and psychological safety operates bidirectionally and in a self-reinforcing manner (Simons & Roberson, 2003; Carmeli & Gittell, 2009). Justice violations — particularly interactional and procedural injustices — signal to employees that the organizational environment punishes rather than protects those who challenge misconduct, thereby directly eroding psychological safety (Nembhard & Edmondson, 2006). Simultaneously, eroded psychological safety silences employees who might otherwise voice corrective information, allowing injustices to persist and intensify (Dollard et al., 2026; Tangirala & Ramanujam, 2008). This reciprocal dynamic generates either virtuous cycles (justice → safety → voice → maintained justice) or vicious cycles (injustice → fear → silence → perpetuated injustice).

*Cross-Cultural Validity of the Theoretical Frameworks:* A legitimate concern exists about the transferability of theories developed predominantly in Western organizational contexts to non-Western settings. Regarding Organizational Justice Theory, Leake et al. (2025), Brockner et al. (2001), and Leung et al. (2002) provide compelling cross-cultural evidence that justice principles — particularly procedural and interactional — retain explanatory power across high- and low-power-distance cultures. What culture appears to alter is not whether fairness matters, but how fairness is enacted and read: which behaviors are recognized as violations in the first place, how serious a violation must become before employees name it, and which responses are socially permissible once it has been named. Regarding Psychological Safety Theory, Frazier et al.’s (2017) multi-country meta-analysis confirmed the theory’s cross-cultural applicability, while research in Asian and Middle Eastern contexts (Lian et al., 2012; Al-Kahtani et al., 2021) has shown that psychological safety dynamics operate similarly across collectivist, high power-distance settings. Evidence from Saudi organizations points in the same direction: employees there evaluate leaders’ conduct against fairness criteria that mainstream constructs describe well, even where the idiom in which those judgments are voiced remains locally specific (Mohiya, 2023). The current study, therefore, treats both frameworks as culturally portable while acknowledging that their specific manifestations are shaped by Saudi Arabian organizational culture, Islamic values, and collectivist relational norms — an approach consistent with universalist-contextual theoretical integration (Gelfand et al., 2011).

### 2.5 Empirical Literature on Mistreatment Behavior

#### 2.5.1 Prevalence and Consequences

Empirical evidence documents the widespread prevalence of mistreatment behavior across organizational contexts. Abusive supervision affects an estimated 13.6% of U.S. employees (Schat et al., 2006), while estimates of incivility are considerably higher, ranging from 75% to 98% across sectors (Cortina et al., 2013). Manifestations include verbal aggression (Neuman & Baron, 1998), abusive supervision (Tepper, 2007), systematic bullying (Einarsen et al., 2020), and discrimination (Goldman et al., 2006). Victims experience elevated psychological distress, anxiety, emotional exhaustion, and compromised physical health (Mensah et al., 2026; Bowling & Beehr, 2006; Nielsen & Einarsen, 2012), alongside diminished satisfaction, commitment, and performance, and heightened turnover intentions (Hershcovis & Barling, 2010). More recent evidence extends these findings: Liang et al. (2022) suggested that abusive supervision is dynamically self-perpetuating through supervisor self-regulation failure, while Mackey et al. (2021) conducted a meta-analysis confirming its destructive effects on multiple performance and well-being outcomes. Han et al. (2022) further documented how abusive supervision degrades employees’ sleep, emotional states, and creative output. The corollary is well established: the psychological and relational conditions employees encounter at work are among the strongest determinants of whether they contribute ideas at all (Khan & Mohiya, 2020).

#### 2.5.2 Power, Hierarchy, and Supervisory Mistreatment

Research consistently identifies power asymmetries as structurally central to mistreatment dynamics. Individuals with formal authority command disproportionate capacity to inflict harm while facing attenuated accountability (Tepper et al., 2017). Supervisory control over career-consequential outcomes — performance ratings, advancement opportunities, task assignments — intensifies subordinates’ vulnerability and narrows the available repertoire of responses (Tepper, 2007; Mitchell & Ambrose, 2012). Employees’ assessments of those in authority are, moreover, formed and circulated well beyond formal channels, including on the organization’s own internal platforms, where leader conduct is discussed, compared, and appraised (Mohiya, 2025c).

#### 2.5.3 Barriers to Reporting and Voice

Cortina and Magley (2009) found that only approximately 30% of harassment victims pursue formal complaints, with anticipated retaliation functioning as the predominant deterrent (Near & Miceli, 2016). Employees calculate that reporting risks poor performance evaluations, blocked career advancement, or outright termination — particularly when perpetrators occupy powerful positions (Milliken et al., 2003). Anticipated futility — the belief that complaints will be dismissed or ignored — constitutes an additional powerful inhibitor (Morrison, 2014). In collectivist cultural settings, the prospect of being labeled a ‘troublemaker’ who disrupts group harmony intensifies reluctance to engage formal reporting channels (Gelfand et al., 2007). Trust in the system through which a concern must travel is itself a precondition of its use; where that trust is absent, employees withdraw from the channel rather than from the concern (Mohiya, 2025d).

#### 2.5.4 Cultural Contexts and Mistreatment Dynamics

Cross-cultural organizational research documents substantial variation in behavioral norms and employee response dispositions (Aycan, 2005; Gelfand et al., 2011). Power distance — the extent to which less powerful organizational members accept hierarchical inequality as legitimate (Hofstede, 2001) — is particularly theoretically salient, with high power distance cultures generally exhibiting stronger tendencies toward hierarchical deference and acceptance of directive leadership (Pellegrini & Scandura, 2008; Brockner et al., 2001). Collectivist values emphasizing relationship preservation and face-saving compound reporting inhibitions by adding cultural transgression dimensions to the calculation of interpersonal risk (Morrison, 2011; Gelfand et al., 2007). Despite its economic prominence, research on organizational behavior in the Middle East remains severely underrepresented in mainstream scholarship (Mellahi & Budhwar, 2010), a gap that this study directly addresses and to which qualitative inquiry inside Saudi organizations has recently begun to contribute (Mohiya, 2023).

#### 2.5.5 Employment Precarity and Differential Vulnerability

Emerging scholarship examines how employment status categories produce systematically differential vulnerability to mistreatment (Liden et al., 2003). Non-permanent, agency-affiliated, or contract workers frequently encounter second-class treatment, social exclusion, and constrained access to protective mechanisms (Van Dyne & Ang, 1998; Gallagher & McLean Parks, 2001). Employment precarity amplifies mistreatment exposure while simultaneously constraining employees’ capacity to voice concerns or seek redress, creating a particularly damaging nexus of vulnerability.

## 3. Research Design and Methodology

### 3.1 Philosophical Positioning and Research Approach

This investigation employs a qualitative, exploratory, theory-informed single-case study design (Yin, 2018), oriented toward generating a rich, contextually situated understanding of the dynamics of mistreatment behavior within a large Saudi Arabian multinational organization. This design choice is appropriate for examining complex, context-embedded phenomena whose meaning cannot be abstracted from their organizational and cultural settings (Stake, 1995; Flyvbjerg, 2011). Single-case and qualitatively triangulated designs of this kind have proved productive in earlier investigations of employee experience in Saudi settings (Mohiya, 2024a, 2024b, 2026f).

Philosophically, the study operates within a social constructivist epistemological tradition (Berger & Luckmann, 1966), treating individual and collective meaning-making as constitutive of organizational reality. Simultaneously, a critical realist ontological commitment (Bhaskar, 1978) is maintained, acknowledging that although interpretations are socially produced, underlying structural mechanisms — hierarchical power, institutional arrangements, cultural norms — exert causal influence on employees’ experiences independent of individual perception (Danermark et al., 2002). This dual positioning enables the study to honor phenomenological complexity while retaining analytical traction on structural dynamics. This dual ontological-epistemological positioning is consistent with recent qualitative organizational scholarship (Cunliffe, 2011; Creswell & Poth, 2018) and has been deployed effectively in comparable studies of workplace behavior (Lutgen-Sandvik & Tracy, 2012; Tracy, 2020).

### 3.2 Research Setting

The research was conducted within a large multinational corporation headquartered in Saudi Arabia, which operates in the energy and industrial sectors and employs more than 60,000 people. The organization encompasses extensive international operations and employs a highly heterogeneous workforce comprising Saudi nationals, Arab expatriates, South and Southeast Asian professionals, and Western specialists. It publicly articulates strong commitments to professional management, corporate integrity, and respect-based workplace culture.

*Case Boundaries:* The case is bounded by: (1) organizational scope — all data were collected from employees working within the organization’s Saudi Arabian operations; (2) temporal scope — data were generated between 2014 and 2020; (3) substantive scope — the case concerns the organizational phenomenon of mistreatment behavior as experienced and interpreted by employees. The study does not extend to customers, external stakeholders, or employees in the organization’s international operations outside Saudi Arabia. These explicit boundaries are consistent with Yin’s (2018) guidelines for single-case study design.

The organization was selected through purposive sampling based on: (1) sufficient scale and structural complexity to generate diverse and differentiated mistreatment experiences; (2) workforce heterogeneity enabling analysis of nationality, employment category, and cultural background as contextual conditions; (3) the existence of formal policies explicitly prohibiting workplace misconduct; (4) researcher access to organizational data; and (5) representativeness for large Middle Eastern organizations navigating modernization pressures while maintaining hierarchical structures.

*Transparency Regarding Case Selection:* The selection of this particular organization may have been driven more by data availability (access to the internal communication platform) than by a systematic comparison of candidate organizations. I acknowledge this openly. The case was identified through the primary researcher’s professional access to the organization’s internal platform, and no formal multi-candidate comparison process was conducted. The five selection criteria (size, diversity, formal policies, data access, representativeness) are applied retrospectively as justificatory criteria rather than as a prospective selection algorithm. This represents a genuine limitation of the research design. The case is treated as a theoretically purposive rather than systematically comparative selection, and the study’s findings should be interpreted accordingly — as generating theoretical propositions for future multi-case testing rather than as representative of all large Middle Eastern multinationals.

### 3.3 Data Sources and Collection Procedures

#### 3.3.1 Internal Communication System Comments

Integration of Temporally Distinct Data Sources: The study triangulates two data streams collected at different points in time — internal communications (2014) and semi-structured interviews (2017–2020). The study’s design logic justifies this integration. Both sources are treated as windows onto the same underlying organizational phenomenon (mistreatment dynamics within a specific organization), rather than as measurements of the same variables at different time points. The gap between sources is acknowledged as a limitation (see Section 6.4) but does not invalidate triangulation, which is warranted by the high thematic convergence observed. The interview data serve a temporal updating function, confirming that the patterns evident in the 2014 blog data remained organizationally relevant several years later. This convergence supports the interpretation that the documented dynamics reflect structurally embedded organizational features rather than transient episodes.

The primary data corpus comprises 197 employee-generated comments, extracted from the organization’s internal communication platform, in response to a formal post addressing workplace mistreatment. This platform, accessible to all permanent and contract employees, functions as a channel for HR communications, policy dialogues, and employee-generated discourse. The target post explicitly solicited employee perspectives regarding experiences with, perceptions of, and suggestions concerning mistreatment behavior. Comments were posted across six months.

*Temporal Representativeness of the Blog Data:* The blog comments were generated in 2014, pre-dating the interviews (2017–2020) by several years. I acknowledge this temporal gap and recognize it as a methodological limitation warranting transparency. However, several considerations support the continued relevance of these data. First, the organizational phenomena identified — hierarchical coercion, fear of retaliation, nepotism, employment status discrimination, and the policy-practice gap — reflect deeply embedded structural and cultural patterns unlikely to have transformed fundamentally within the subsequent decade. Second, the interview data (2017–2020) provide a temporal update, and the high thematic convergence between the two sources supports the stability of the identified organizational dynamics. Third, large, complex organizations of this type are well documented to exhibit significant institutional inertia (Hannan & Freeman, 1984). Nonetheless, readers should be aware that direct temporal comparison between the two data sources was not the study’s intention; rather, both sources are treated as complementary streams of triangulation illuminating the same underlying organizational phenomenon over a period of sustained organizational activity.

Naturally occurring digital discourse of this kind offers distinctive methodological advantages over researcher-elicited data: it captures employees’ spontaneous, self-selected expressions without the mediating influence of interviewers or the social desirability pressures inherent in researcher-initiated encounters. These data represent authentic organizational discourse, revealing the language, conceptual frameworks, and priority concerns employees deploy when addressing their colleagues in a semi-public organizational forum (Kozinets, 2015; Pink et al., 2022). The evidentiary standing of such platforms has been examined directly in earlier work: employees read organizational postings as institutional signals about what an organization values and tolerates (Mohiya, 2026b); they use internal human resource platforms selectively and strategically (Mohiya, 2026a, 2026g); and platform design and functionality shape which contributions become visible and which do not (Mohiya, 2025f). Employee engagement with enterprise social networking in a large Saudi energy organization has itself been the subject of a separate, earlier investigation (Mohiya, 2019).

*Transparency Regarding Organizational Mediation:* I acknowledge that this internal platform was accessible to all employees, including HR staff and line management, creating a context meaningfully different from fully private discourse. This semi-public visibility likely introduced a degree of self-censorship, as employees would reasonably consider who might read their comments. This represents a genuine limitation of this data source, which is explicitly addressed in Section 6.4. At the same time, the platform’s accessibility to all employees meant comments were made voluntarily in a setting employees knew was organizationally visible — arguably rendering the concerns expressed more salient and considered, rather than less so, since contributors knowingly chose to express them. Nonetheless, readers should interpret these data as filtered rather than entirely naturalistic organizational discourse, and the study’s triangulation with private, semi-structured interviews (in a more confidential setting) partially compensates for this limitation.

#### 3.3.2 Semi-Structured Interviews

The complementary data source comprises thirty-nine semi-structured individual interviews with employees, conducted across multiple organizational levels, functions, employment categories, and national backgrounds between 2017 and 2020. Interviews ranged from 45 to 90 minutes in duration, were audio-recorded with participants’ informed consent, and subsequently transcribed verbatim and subjected to member checking.

The interview protocol incorporated open-ended exploratory questions examining: (1) employees’ conceptualizations of mistreatment behavior; (2) personal experiences of or exposure to mistreatment; (3) perceived organizational responses to reported misconduct; (4) factors influencing decisions to voice or remain silent; (5) perceived drivers and enablers of mistreatment; and (6) cultural factors shaping behavioral expectations and responses. The semi-structured format allowed systematic coverage of conceptually anchored topics while preserving flexibility to pursue emergent analytical threads (Brinkmann & Kvale, 2015).

### 3.4 Sampling and Participant Profiles

The 197 internal communication comments constitute a self-selected convenience sample. Complete demographic data were unavailable for all contributors; the platform did not systematically collect or attach demographic identifiers to posts. What is known contextually is that the platform was accessible to all permanent and contract employees across the organization’s Saudi Arabian operations, and that the thread generated sustained engagement over six months, suggesting contributors represented diverse organizational constituencies. However, I explicitly acknowledge that this constitutes a convenience sample of self-selecting employees, and representativeness cannot be verified or guaranteed. Specifically, I recognize that employees experiencing mistreatment may be overrepresented, and — critically — that within the group of affected employees, those experiencing the most severe fears of retaliation may be systematically underrepresented, since such employees are precisely those least likely to post in a semi-public forum. Readers should interpret findings from the blog data with this dual selection bias in mind and use their own judgment about the degree of representativeness. The interview data, collected in private settings with confidentiality guarantees, provide a complementary dataset less subject to these specific biases.

Interview participants were recruited through a combination of purposive and snowball sampling strategies (Patton, 2015), designed to ensure representation across gender, nationality, employment category, organizational level, and tenure—see Table 1 below for participant demographics.

**Table 1:** Semi-Structured Interview Participant Profiles (N = 39).


CHARACTERISTIC	CATEGORY	N	%

**Gender**	Male	26	66.7

	Female	13	33.3

**Nationality**	Saudi National	15	38.5

	Arab Expatriate (non-Saudi)	10	25.6

	South/Southeast Asian	9	23.1

	Western (European/North American)	5	12.8

**Employment Category**	Permanent/Direct Employee	24	61.6

	Fixed-Term Contract	10	25.6

	Agency/Outsourced	5	12.8

**Organizational Level**	Operations/Technical Staff	13	33.3

	Professional/Senior Specialist	16	41.1

	Team Leader/Middle Manager	7	17.9

	Senior Manager/Director	3	7.7

**Years of Organizational Tenure**	Fewer than 3 years	6	15.4

	3 to 7 years	12	30.8

	8 to 14 years	13	33.3

	15 years or more	8	20.5

**Highest Educational Qualification**	Diploma or Vocational Certificate	5	12.8

	Bachelor’s Degree	19	48.7

	Postgraduate (Master’s/PhD)	15	38.5


*Note*. N = 39. Percentages are rounded to one decimal place. Within Employment Category and Organizational Level, rounding leaves a residual of 0.1; this has been absorbed into the largest category in each series so that every series sums to 100.0.

*Sampling Adequacy and Thematic Saturation:* Sampling continued until thematic saturation was reached — the point at which additional interviews yielded no substantively new codes or themes (Guest et al., 2006). Saturation was assessed iteratively: after the 30th interview, no new first-order codes were generated. Interviews 31–39 served as a confirmatory phase, validating the stability of the thematic structure. This process yielded the five-theme structure presented in Section 4, which I consider adequately saturated, given the purposive sampling strategy that targets maximum diversity across key characteristics.

### 3.5 Analytical Approach: Reflexive Thematic Analysis

Data were analyzed using Braun and Clarke’s (2006, 2019) reflexive thematic analysis framework, which provides a systematic, philosophically coherent, and flexible methodology for identifying, interpreting, and reporting patterns of meaning within qualitative data. This approach accommodates both inductive (data-driven) and deductive (theory-informed) coding orientations (Braun & Clarke, 2012; Terry et al., 2017).

*Phase 1 (Familiarization):* Immersive, repeated engagement with the entire dataset enabled initial intuitive sense-making and notation of preliminary analytical observations.

*Phase 2 (Initial Code Generation):* Systematic line-by-line coding was applied across both data sources, generating deductive codes anchored in theoretical constructs (e.g., ‘procedural injustice,’ ‘fear of retaliation’) alongside inductive codes arising organically from the data (e.g., ‘performance rating weaponization,’ ‘agency worker dehumanization’). NVivo 12 was employed for data management. The coding process involved two researchers: the primary researcher (the author) and an independent research collaborator with expertise in qualitative organizational research. Both researchers independently coded a stratified random subsample of 40% of the dataset (approximately 79 internal communications and 16 interviews). Inter-coder reliability was assessed using Cohen’s kappa, yielding κ = 0.81, indicating strong agreement (Landis & Koch, 1977). Disagreements were resolved through discussion and consensus. The remaining data were coded by the primary researcher, with periodic audit checks by the collaborator.

*Phase 3 (Theme Construction):* Initial codes were clustered and reorganized into candidate thematic categories representing broader, analytically coherent patterns of meaning.

*Phase 4 (Thematic Review and Refinement):* Candidate themes were iteratively assessed against the full dataset to ensure internal coherence and meaningful differentiation between themes (Patton, 2015).

*Phase 5 (Theme Definition and Naming):* Each theme was precisely defined and bounded, its scope articulated, and its relationship to the study’s research questions and theoretical framework specified.

*Phase 6 (Report Production):* The final analytic narrative integrates themes with theoretical elaboration, illustrative quotations, and cross-data source triangulation.

### 3.6 Quality, Rigor, and Trustworthiness

Credibility was strengthened through data source triangulation (internal communications and interviews), prolonged researcher engagement with the organizational context, member checking of interview interpretations with participants, and peer analytic debriefing. Transferability is supported by thick, contextually detailed descriptions and the theoretically grounded analytical framework that facilitates principled extrapolation to comparable settings. Dependability was assured through a systematic audit trail, multi-coder involvement, inter-rater reliability assessment, and adherence to transparent analytic procedures. Confirmability was promoted through explicit reflexive positioning, systematic grounding of interpretations in verbatim data, and avoidance of unsupported inferential leaps (Lincoln & Guba, 1985; Guba & Lincoln, 2005). To provide more than a nominal checklist, the following procedural specifics are offered: (1) Member checking involved sharing thematic summaries with six interview participants drawn purposively from different organizational levels and nationalities; all confirmed the resonance of the themes with their experiences, and two offered minor clarifications that refined the articulation of Theme E. (2) Peer debriefing involved two iterative sessions with a senior qualitative researcher external to the project, who challenged analytical interpretations and identified one instance of overgeneralization in the early draft of the model. (3) Thick description is embedded throughout Section 4 through verbatim quotation and contextual commentary, providing readers with sufficient grounding to assess transferability independently.

### 3.7 Ethical Considerations

This study was conducted with the approval of the Deanship of Scientific Research Ethics Committee (approval reference: ECM#2026-1101) and in strict accordance with the Declaration of Helsinki (2013). All interview participants provided fully informed written consent before participation. Participants were explicitly guaranteed the right to voluntary participation, unrestricted withdrawal rights, complete anonymity, and exclusive use of data for academic purposes. Internal communication data were treated as secondary organizational data; all identifying information was systematically anonymized before analysis, and data were stored in encrypted, access-controlled repositories accessible exclusively to the research team. The approval reference number (ECM#2026-1101) is consistently reported both here and in the Declarations section.

*Ethical Considerations for Blog Data:* The ethical treatment of the internal communication data requires specific justification. Employees posting on the internal platform could reasonably expect their comments to be read by colleagues, HR staff, and management — the platform’s explicit function was to facilitate organizational dialogue. In this sense, while employees did not expect full privacy, they did have a reasonable expectation that their comments would serve an internal organizational rather than an external research purpose. To address this ethical complexity: (1) all comments were fully anonymized before analysis, with organizational roles, names, and identifiable contextual details removed; (2) the research received explicit ethical approval covering the use of this data type; (3) no attempt was made to re-identify contributors; and (4) verbatim quotations presented in the manuscript are presented at a level of generality that prevents identification. I acknowledge the ethical tension between using data posted for organizational rather than research purposes, and I have sought to handle it with the care that this complexity demands.

Formal organizational access was negotiated with the organization’s senior management and HR leadership before the commencement of the interview phase. Management was informed of the research’s academic purpose, the voluntary nature of participation, and the strict confidentiality protections applied to all data. Organizational gatekeepers facilitated recruitment through internal communication channels, though they had no visibility into which individual employees chose to participate, nor into the content of interviews. This arrangement balanced organizational access requirements with the independence necessary for authentic participant disclosure.

## 4. Findings

Reflexive thematic analysis of the combined dataset yielded five overarching thematic categories that collectively illuminate the experiential landscape of mistreatment behavior in the research setting, and its implications for organizational justice, psychological safety, and employee voice (see Table 2).

**Table 2:** Thematic Categories, Sub-Themes, and Empirical Prevalence.


THEMATIC CATEGORY	SUB-THEMES IDENTIFIED	PROPORTION OF CODED DATA (%)	EVIDENCE SOURCES

**Theme A: Authority Misuse and Supervisory Coercion**	A1: Intimidation and Verbal Hostility; A2: Coercive Performance Leverage; A3: Exclusionary Leadership Practices; A4: Promotion-Induced Behavioral Regression	32%	Internal Communications: 83 entries; Interviews: 36 participants

**Theme B: Suppression of Employee Voice and Retaliatory Culture**	B1: Fear of Professional Reprisal; B2: Informal Intimidation Networks; B3: Cynicism Toward Reporting Mechanisms; B4: Collective Withdrawal from Disclosure	27%	Internal Communications: 71 entries; Interviews: 37 participants

**Theme C: Structural and Procedural Inequities**	C1: Employment-Category-Based Discrimination; C2: Nepotism and Informal Influence Networks; C3: Selective Enforcement of Organizational Policies; C4: Absence of Grievance Transparency	20%	Internal Communications: 62 entries; Interviews: 32 participants

**Theme D: Organizational Betrayal and Eroded Institutional Confidence**	D1: Dissonance Between Stated Values and Enacted Conduct; D2: Inadequate Enforcement of Anti-Mistreatment Pledges; D3: Organizational Shielding of Offenders; D4: Entrenched Disillusionment and Disengagement	14%	Internal Communications: 48 entries; Interviews: 27 participants

**Theme E: Socio-Cultural and Institutional Conditions**	E1: Hierarchical Deference vs. Dignity Expectations; E2: Harmony-Oriented Social Norms and Silence; E3: Religious-Ethical Frameworks as Normative Anchors; E4: Generational Shifts in Workplace Expectations	7%	Internal Communications: 33 entries; Interviews: 21 participants


*Cross-Source Convergence:* A structured comparison of findings across the two data sources — internal communications and semi-structured interviews — reveals high thematic convergence, strengthening confidence in the study’s credibility. All five themes were independently evident in both data sources. Theme A (Authority Misuse) was most prominent across both the blog (83 entries, 42% of coded blog data) and interviews (36 of 39 participants). Theme B (Voice Suppression) showed the strongest interview prevalence (37 of 39 participants), while its blog footprint was somewhat lower (71 entries, 36%), consistent with the prediction that the most retaliation-fearing employees would be underrepresented in the semi-public blog. This differential pattern constitutes meaningful evidence for the study’s central argument: the very employees most subject to silencing mechanisms were comparatively underrepresented in the more visible data source. Theme E (Cultural Conditions) was more richly articulated in interviews (21 participants) than in the blog (33 entries), consistent with the greater depth of interview data. These cross-source patterns were examined descriptively; formal statistical testing between qualitative coded themes is not methodologically appropriate or epistemologically aligned with the study’s constructivist framework, though the convergence pattern is analytically informative.

### 4.1 Theme A: Authority Misuse and Supervisory Coercion (32%)

The most prominent thematic category captured systematic patterns of supervisory power misuse, manifested through verbal hostility, intimidatory conduct, and the deliberate manipulation of evaluation systems for coercive ends. These behaviors constituted compounded violations of interactional justice intensified by structural power asymmetry.

#### 4.1.1 Intimidation and Verbal Hostility

Shouting, verbally demeaning language, and openly hostile communication directed at subordinates emerged as the most recurrently reported conduct across both data sources:

*Managers routinely use degrading language when addressing employees and leverage their positional authority to issue threats — we cannot escalate this because we cannot afford to make enemies in the workplace*. (Internal Communications)

*When my supervisor shouts at me in front of colleagues, the humiliation goes beyond the words. It signals to everyone that I can be treated as worthless. Moreover, you cannot respond because he determines your rating and your future here*. (Interview P-21)

These accounts reflect unambiguous violations of interactional justice — specifically, interpersonal justice norms mandating dignified, courteous treatment (Bies & Moag, 1986). The public character of such conduct intensifies its injurious effects by compounding the immediate emotional harm with reputational degradation (Mensah et al., 2026; Bies, 2015; Liang et al., 2022).

#### 4.1.2 Coercive Performance Leverage

Employees across multiple organizational grades described explicit linkages between compliance and performance evaluation outcomes:

*My direct manager made it unmistakably clear that unless I agreed to weekend work without additional compensation, my performance rating would suffer. He stated this directly in a one-to-one meeting*. (Interview P-29)

Such conduct constitutes a severe violation of interactional justice while simultaneously eroding psychological safety by signaling that organizational environments actively punish interpersonal risk-taking (Edmondson, 1999; Mitchell & Ambrose, 2012). It also inverts the logic of a performance-driven culture, which depends on employees volunteering discretionary knowledge and effort rather than surrendering them under threat (Mohiya, 2023).

#### 4.1.3 Exclusionary Leadership Practices

Multiple participants reported systematic exclusion from decision-making processes, developmental opportunities, and information flows as a coercive leadership practice:

*Employees who asked questions or raised concerns were quietly excluded from key meetings, training nominations, and their ideas were attributed to others. It was a deliberate marginalization strategy*. (Interview P-14)

Such exclusionary practices extend mistreatment beyond overt aggression into more diffuse but equally damaging forms of organizational mistreatment (Cortina et al., 2013; Hershcovis, 2011).

#### 4.1.4 Promotion-Induced Behavioral Regression

A recurrent narrative concerned behavioral deterioration following supervisory promotion:

*I worked alongside this individual for four years as peers. After the promotion, he became unrecognizable — dismissive, publicly critical, and took credit for others’ contributions. Power appears to have no accountability mechanism here*. (Interview P-11)

These narratives suggest that organizational power structures and inadequate supervisory preparation create conditions that enable behavioral regression, consistent with evidence of power’s corrupting effects in the absence of accountability (Kipnis, 1972; Tepper et al., 2017). They also underline how far employees’ daily experience of the organization is manufactured at the frontline and middle-management layer rather than at the level of corporate policy (Mohiya, 2025a).

### 4.2 Theme B: Suppression of Employee Voice and Retaliatory Culture (27%)

The second thematic category documented pervasive, fear-driven organizational silence in which employees systematically withheld concerns about mistreatment, not from indifference, but from rational anticipation of adverse consequences — directly engaging psychological safety theory.

#### 4.2.1 Fear of Professional Reprisal

Anticipated career consequences constituted the dominant barrier to voice:

*What is at risk if you report your direct supervisor? Your performance rating, your promotion trajectory, your professional reputation, your access to development opportunities — everything. The calculation is simple and brutal*. (Internal Communications)

*A colleague formally reported verbal abuse by our supervisor. The supervisor faced no consequence whatsoever. But my colleague? Within a month, every minor procedural deviation was formally documented. His annual rating dropped significantly. He was overlooked for promotion twice in succession. The organizational message could not have been more explicit*. (Interview P-12)

These accounts demonstrate exactly the rational fear-suppression dynamic theorized by Morrison (2014) and Milliken et al. (2003): employees engage in cost-benefit analyses and conclude that the personal costs of voice substantially exceed anticipated benefits (Near & Miceli, 2016).

#### 4.2.2 Informal Intimidation Networks

Beyond formally enacted retaliation, employees described informal social mechanisms sustaining silence:

*In this organizational culture, lodging a formal complaint against a supervisor — even a justified one — transforms you into the identified problem. You are labeled as someone who cannot be managed, who creates disruption. This social stigmatization persists regardless of the formal outcome. So people choose to absorb mistreatment rather than bear that label*. (Interview P-20)

The ‘making enemies’ concern reflects collectivist cultural values prioritizing relational harmony and face-maintenance (Gelfand et al., 2007), which compound psychological safety deficits by adding cultural transgression to the calculus of interpersonal risk-taking (Hofstede, 2001).

#### 4.2.3 Cynicism Toward Reporting Mechanisms

Many employees articulated deep beliefs that complaints produce no meaningful organizational response:

*There are numerous complaints about all categories of employee mistreatment. The fundamental reality is that these concerns reach no decision-making authority. No one with the power to act is listening*. (Internal Communications)

*Multiple individuals in my network have submitted formally structured complaints through designated channels. What occurred? Individuals with collegial relationships to the accused investigated each complaint. Nothing changed structurally. The complainants acquired reputational difficulties*. (Interview P-16)

Perceived futility produces organizational cynicism (Dean et al., 1998). It reinforces silence, representing a compounded failure of both procedural justice (manifestly ineffective systems) and psychological safety (absence of assurance that voice is protected) (Morrison, 2014).

#### 4.2.4 Collective Withdrawal from Disclosure

Employees described the emergence of collectively shared, socially transmitted beliefs that reporting is structurally futile:

*No one in my department reports anything. Not because no one has experienced mistreatment — everyone has. However, because everyone has witnessed or heard about cases in which reporting resulted in professional harm for the reporter and no consequence for the perpetrator. Collective silence has become the rational adaptation*. (Interview P-22)

This collective withdrawal exemplifies systemic organizational silence (Morrison & Milliken, 2000): a climate-level phenomenon shaped by vicarious learning from observed punishment of voice (Bandura, 1977), representing a profound failure of psychological safety at the organizational level.

### 4.3 Theme C: Structural and Procedural Inequities (20%)

Beyond interpersonal mistreatment, employees identified deeply embedded organizational arrangements that generate systemic unfairness and compound individual-level experiences of injustice.

#### 4.3.1 Employment-Category-Based Discrimination

Systematic differential treatment predicated on employment classification represented one of the most frequently and vehemently described justice violations:

*Supervisors in senior positions routinely treat non-permanent employees as if they occupy a fundamentally lower human category. The dehumanization is not incidental — it appears to be a structurally expected entitlement of the supervisory role*. (Internal Communications)

*The discrimination pervades every dimension of organizational life: entry procedures, facility access, equipment quality, supervisory tone, training nomination priorities, everything. We are performing work that is equivalent to, or more technically complex than, yet we are constantly reminded of our subordinate organizational status*. (Interview P-09)

*The most insidious aspect is the power asymmetry created by employment insecurity. When agency-contracted employees are subjected to supervisory mistreatment, their realistic options for response are essentially zero. Formal reporting risks immediate contract termination. The vulnerability is systematic and deliberately exploited*. (Interview P-37)

Employment status discrimination constitutes compounded distributive injustice (inequitable allocation of dignity, access, and opportunity based on categorical rather than merit-based criteria) and interactional injustice (systematic violation of dignity norms) (Colquitt, 2001). This structural caste-like dynamic creates differential vulnerability at the intersection of employment precarity and power asymmetry (Liden et al., 2003; Gallagher & McLean Parks, 2001).

#### 4.3.2 Nepotism and Informal Influence Networks

Participants identified nepotism — locally conceptualized through the Arabic term ‘wasta,’ denoting the strategic deployment of personal connections — as a pervasive distributive and procedural justice violation:

*Tribal affiliation, kinship networks, and national group membership determine advancement, assignment quality, and supervisory treatment. Technical competence and committed effort are rendered largely irrelevant. This generates profound organizational resentment and destroys any meaningful belief in meritocracy*. (Interview P-13)

Favoritism constitutes simultaneous violations of distributive justice (allocations that violate equity principles) and procedural justice (decision-making processes that lack consistency and fail to suppress bias) (Leventhal, 1980; Cropanzano et al., 2017). Systemic favoritism signals that organizational systems are fundamentally corrupted, generating widespread cynicism and comprehensively eroding collective justice climates (Ambrose & Schminke, 2009).

#### 4.3.3 Selective Enforcement of Organizational Policies

Employees observed that anti-mistreatment policies were applied differentially based on organizational position:

*When frontline employees commit procedural errors, consequences follow promptly and visibly. When supervisors engage in verbally abusive conduct that multiple witnesses document, nothing happens. The existence of a hierarchical double standard is not implied — it is explicitly demonstrated*. (Interview P-15)

Inconsistent policy application violates procedural justice standards of consistency and bias suppression (Leventhal, 1980), communicating to employees that rules protect power rather than principle (Colquitt et al., 2013).

#### 4.3.4 Absence of Grievance Transparency

Employees articulated procedural justice concerns about the opacity of misconduct reporting mechanisms:

*The most fundamental questions have no accessible answers. To whom does one report? What investigative process applies? How are confidentiality obligations enforced? What constitutes actionable evidence? Without answers to these questions, the formal reporting system is functionally inaccessible, regardless of its nominal existence*. (Interview P-07)

These procedural deficiencies — absence of representativeness, transparency, and corrective mechanisms — constitute comprehensive procedural injustice under Leventhal’s (1980) criteria and Thibaut and Walker’s (1975) framework.

### 4.4 Theme D: Organizational Betrayal and Eroded Institutional Confidence (14%)

This thematic category captured employees’ perceptions of fundamental discrepancies between organizational commitments to dignity and fairness and the institutional realities they experienced, which they regarded as profound violations of the psychological contract.

#### 4.4.1 Dissonance Between Stated Values and Enacted Conduct

The most pervasive expression of institutional betrayal concerned the chasm between espoused organizational values and observable organizational practices:

*The organization’s stated values are genuinely admirable — respect, integrity, dignity, zero tolerance for misconduct. However, they function exclusively as public communication artifacts. The lived organizational reality is that abusive supervisors retain their positions, nepotism governs advancement, and complaints are absorbed without consequence. The dissonance is not subtle*. (Interview P-18)

The policy-practice gap constitutes a breach of the psychological contract — a perceived failure to fulfill implicit and explicit organizational obligations (Robinson & Rousseau, 1994; Morrison & Robinson, 1997). Such breaches generate betrayal, organizational cynicism, disengagement, and erosion of institutional trust (Johnson & O’Leary-Kelly, 2003; Simons, 2002). Employees in Saudi organizations have been shown to evaluate corporate value statements against observed managerial conduct, treating the distance between the two as diagnostic of the organization’s real priorities (Mohiya, 2021).

#### 4.4.2 Inadequate Enforcement of Anti-Mistreatment Pledges

‘Zero tolerance’ commitments were frequently cited as particularly damaging when unimplemented:

*‘Zero tolerance’ as a policy formulation implies an absolute threshold: one verified incident generates an organizational response. Every employee in this organization knows multiple supervisors who have been formally reported on more than one occasion. The continued presence of these individuals is the definitive organizational statement about zero tolerance*. (Interview P-14)

Publicly proclaimed but systemically unenforced policies signal procedural injustice, breach psychological contracts, and, critically, communicate unambiguously that voice is futile — thereby directly destroying the conditions necessary for psychological safety (Edmondson & Lei, 2014).

#### 4.4.3 Organizational Shielding of Offenders

Perhaps the most institutionally damaging pattern involved organizational systems that appeared to protect perpetrators while exposing victims to additional harm:

*I have direct knowledge of three individuals who formally reported abusive supervisors through prescribed organizational channels. All three experienced subsequent professional deterioration — negative performance evaluations, blocked advancement, and lateral transfers to unfavorable positions. All three supervisors remain in post. The organizational logic could not be more explicit about where institutional protection lies*. (Interview P-08)

This pattern represents compounded, multilayered justice violations: procedural (investigation processes exhibiting evident bias), distributive (perpetrators protected while victims are punished), and interactional (treatment of victims that lacks dignity). Such patterns generate profound institutional distrust and clearly signal that psychological safety is unavailable to those who challenge power (Tepper et al., 2017).

#### 4.4.4 Entrenched Disillusionment and Disengagement

Accumulated institutional betrayals produced deeply rooted organizational cynicism:

*I held genuine institutional faith for several years. After sustained observation of the systemic gap between the organization’s stated commitments and its enacted conduct, that faith has been comprehensively eroded. These consultations, surveys, and platforms — they serve communication and public relations functions. They do not alter the underlying structural reality*. (Interview P-19)

Organizational cynicism — a stable, negative evaluative orientation toward the organization characterized by expectations of institutional bad faith — emerges from repeated justice violations, psychological contract breach, and perceived organizational hypocrisy (Johnson & O’Leary-Kelly, 2003; Simons, 2002). It is particularly damaging because it suppresses engagement, organizational citizenship, and any future willingness to provide upward feedback — engagement, after all, being sustained by perceived organizational support and the job satisfaction that such support produces (Mohiya, 2025b).

### 4.5 Theme E: Socio-Cultural and Institutional Conditions (7%)

The final thematic category illuminated how cultural values, religious frameworks, and institutional context shaped both normative expectations regarding workplace conduct and behavioral responses to violations of those expectations.

#### 4.5.1 Hierarchical Deference vs. Dignity Expectations

Participants articulated genuine tension between cultural norms prescribing deference to authority and universally experienced dignity imperatives:

*Our cultural tradition prescribes respect for elders and those in positions of authority. This norm carries significant moral weight. Nevertheless, some supervisors misappropriate it — treating it as a mandate for absolute obedience and interpreting any legitimate question as disrespectful. There is a categorical distinction between cultural deference and acceptance of abuse*. (Interview P-03)

High power distance cultural values (Hofstede, 2001) generate normative expectations of hierarchical deference that may provide enabling conditions for supervisory aggression, while simultaneously coexisting with universal dignity imperatives — which suggests that cultural context conditions how justice concerns are voiced without dissolving them.

#### 4.5.2 Harmony-Oriented Social Norms and Silence

Collectivist cultural orientations, emphasizing group cohesion and face-maintenance, profoundly shaped decisions about voice and silence. The ‘making enemies’ dynamic documented in Theme B reflects precisely these collectivist values: reporting misconduct disrupts relational harmony and attracts the label of organizational troublemaker — concerns that compound psychological safety deficits by adding cultural transgression to interpersonal risk calculations (Gelfand et al., 2007; Morrison, 2011).

#### 4.5.3 Religious-Ethical Frameworks as Normative Anchors

Several participants invoked Islamic ethical principles as moral frameworks for assessing workplace conduct:

*The Islamic tradition commands that every person be treated with inherent dignity, that justice be upheld without exception, and that oppression be actively opposed. When supervisors engage in the behaviors we are discussing, they are not merely violating organizational policies. They are violating foundational religious obligations. This should carry substantial moral weight in a Saudi organization*. (Interview P-05)

Islamic values emphasizing human dignity (karama), justice (adl), and compassionate stewardship (Beekun & Badawi, 2005) provide normative frameworks that explicitly condemn mistreatment. Employees’ invocation of religious authority suggests they seek moral grounds that transcend organizational policy to delegitimize mistreatment.

#### 4.5.4 Generational Shifts in Workplace Expectations

Participants noted emerging generational discontinuities in behavioral expectations and tolerance thresholds, with younger employees socialized within Vision 2030-era institutional reform articulating more assertive expectations regarding participative management, organizational transparency, and workplace dignity. This generational dynamic suggests the normative landscape is in transition, potentially creating conditions for a shift in organizational culture — but also for heightened intergenerational tension in workplaces where older supervisory norms persist (Twenge et al., 2010).

### 4.6 Synthesizing the Findings: The Justice–Safety Vicious Cycle Model

**Figure 1:**
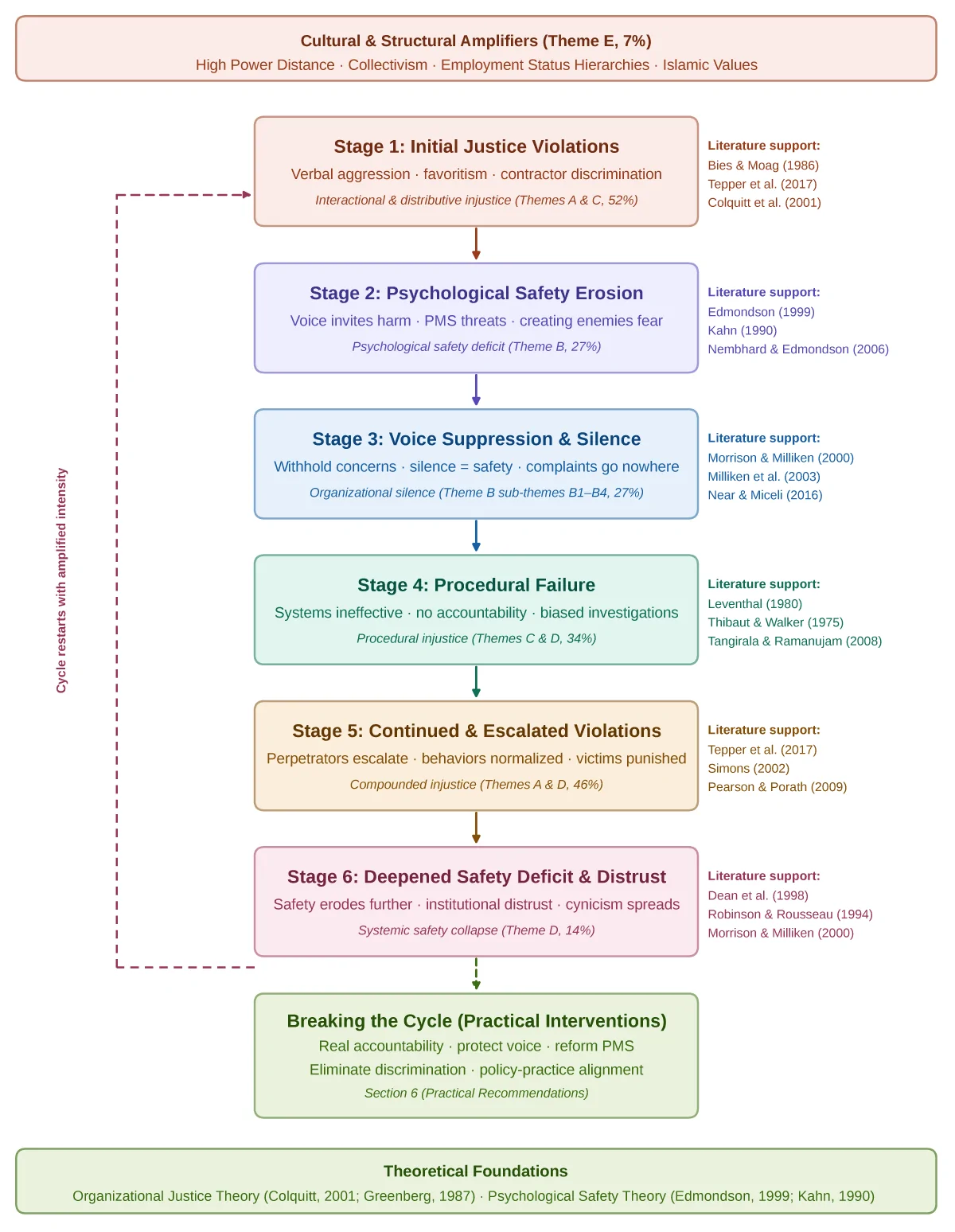
Integrated conceptual framework: the Justice–Safety Vicious Cycle Model. *Note*. OJT = Organizational Justice Theory; PST = Psychological Safety Theory; PMS = performance management system; Themes A–E are defined in Table 2. Solid arrows denote the primary cycle pathway; the dashed arrow denotes the return of the cycle to Stage 1. The model was developed inductively from the reflexive thematic analysis (Braun & Clarke, 2006, 2019) and read against both theoretical frameworks. It is offered as a conceptual framework requiring future quantitative longitudinal testing (see Section 6.5). Percentages give the share of coded data accounted for by the theme or themes grounding each stage; because several themes ground more than one stage, the percentages are not additive.

The Justice–Safety Vicious Cycle Model was constructed through an inductive-deductive process. Each of the six stages emerged directly from the thematic analysis rather than being imposed theoretically: Stage 1 (justice violations) from Themes A and C; Stages 2–3 (safety erosion and voice suppression) from Theme B; Stage 4 (procedural failure) from Themes C and D; Stage 5 (escalated violations) from Themes A and D; Stage 6 (institutional distrust and cynicism) from Theme D. The cyclical return from Stage 6 to Stage 1 is empirically grounded in Theme D sub-theme D4, wherein employees with accumulated distrust explicitly stated they would no longer seek redress for fresh incidents — closing the cycle rather than terminating it linearly. Each stage is cross-validated against Organizational Justice Theory (Colquitt, 2001; Greenberg, 1987) and Psychological Safety Theory (Edmondson, 1999; Kahn, 1990) and is supported by the specific literature cited in Figure 1. The model was iteratively refined through two peer debriefing sessions with an independent qualitative researcher.

Synthesizing Sections 4.1–4.5, the model identifies six mutually implicating stages (see Figure 1): (1) initial justice violations — verbal aggression, favoritism, and contractor discrimination constitute interactional and distributive injustice (Bies & Moag, 1986; Tepper et al., 2017); (2) psychological safety erosion — justice violations signal that environments punish rather than protect dignity (Edmondson, 1999; Kahn, 1990); (3) voice suppression and organizational silence — degraded safety produces systematic withholding of concerns (Morrison & Milliken, 2000; Milliken et al., 2003); (4) procedural failure — absent voice leaves accountability mechanisms dormant, violating consistency and bias-suppression criteria (Leventhal, 1980); (5) continued and escalated violations — unchecked perpetrators normalize misconduct while victims are punished for resistance (Tepper et al., 2017; Pearson & Porath, 2009); and (6) deepened institutional distrust and cynicism — accumulated injustice collapses safety further and restarts the cycle with amplified intensity (Dean et al., 1998; Robinson & Rousseau, 1994). Cultural and structural factors — high power distance, collectivism, employment status hierarchies, and Islamic values (Theme E, 7%) — function as amplifiers at each stage rather than as primary causal agents (Hofstede, 2001; Gelfand et al., 2007).

The model is presented as a conceptual framework that generates testable propositions, not as a statistically verified causal model. Temporal sequencing and directionality require future quantitative longitudinal testing (Section 6.5).

## 5. Discussion

*Study Overview:* This qualitative, single-case exploratory study investigated how 39 interviewees and 197 internal-platform contributors within a large Saudi multinational organization experienced, interpreted, and responded to mistreatment. Drawing on Organizational Justice Theory and Psychological Safety Theory, the study triangulated two complementary data streams to generate five thematic categories: (A) authority misuse and supervisory coercion; (B) suppression of employee voice and retaliatory culture; (C) structural and procedural inequities; (D) organizational betrayal and eroded institutional confidence; and (E) socio-cultural and institutional conditions. The data reveal a complex, mutually reinforcing architecture in which justice violations progressively erode psychological safety, silence perpetuates injustice, and cultural-structural factors amplify each stage of the reinforcement. The discussion below interprets these findings in light of existing theory, identifies extensions and novel contributions, and acknowledges the study’s limitations.

### 5.1 Theoretical Implications for Organizational Justice

The findings robustly support and meaningfully extend Organizational Justice Theory. Consistent with Bies and Moag (1986) and subsequent literature, employees’ most salient concerns centered on interactional injustice—treatment that violated norms of dignity, respect, and courtesy, particularly when enacted by supervisors. These violations generated intense emotional responses and behavioral adaptations, aligning with established justice effects (Mensah et al., 2026; Colquitt et al., 2013). The ‘target similarity principle’ (Rupp & Cropanzano, 2002) is confirmed: supervisor-directed interactional injustice proved disproportionately harmful.

The research extends justice theory by demonstrating compounding justice effects. Rather than experiencing justice dimensions in discrete ways, employees encountered simultaneous, interacting injustices whose combined harm exceeded that of any single dimension: initial interactional violations (verbal aggression) were compounded by distributive violations (rewards to perpetrators, penalties for victims) and procedural violations (biased investigations, inconsistent enforcement). This finding argues for configurational approaches examining justice profiles rather than isolated dimensional effects in future research.

A novel theoretical contribution concerns the identification of procedural justice mechanisms as part of the psychological safety infrastructure. Fair procedures that meet Leventhal’s (1980) criteria serve not merely to generate direct fairness perceptions but also to signal whether environments safely accommodate voice. When complaint processes operate inconsistently and fail to protect complainants, they signal both procedural unfairness and the futility and danger of speaking up. This dual signaling function suggests organizations cannot meaningfully decouple procedural justice design from psychological safety cultivation — they are systemically intertwined. This integration enriches both theoretical literatures.

Regarding cross-cultural applicability, findings both support and nuance existing theory. While some participants acknowledged cultural norms of hierarchical deference that might normalize certain supervisory latitude, the substantial majority condemned mistreatment conduct across all cultural backgrounds by appeal to universal dignity principles and Islamic ethical frameworks. This pattern supports arguments for the cross-cultural relevance of fundamental justice principles (Leung et al., 2002; Brockner et al., 2001) while confirming that cultural context shapes the expression, thresholds, and response repertoires of mistreatment. The research also reveals that justice theory retains analytical traction in non-Western collectivist contexts, though it requires contextually sensitive application.

### 5.2 Theoretical Implications for Psychological Safety

The findings powerfully confirm the centrality of psychological safety to understanding mistreatment dynamics (Edmondson, 1999; Morrison & Milliken, 2000). Employees suppressed their voices not out of disengagement or indifference, but out of rational assessments that disclosure would trigger disproportionate adverse consequences. The specific constellation of barriers identified — career retaliation fears, informal social sanctions, anticipated futility, deep institutional distrust — aligns precisely with established voice-suppression mechanisms (Milliken et al., 2003; Morrison, 2014; Frazier et al., 2017).

The research identifies performance management weaponization as a theoretically distinctive and practically significant destroyer of psychological safety. By controlling career-consequential outcomes — bonus allocations, promotion decisions, training access, employment continuation — supervisors possess extraordinary coercive leverage over voice behavior. This finding extends psychological safety theory beyond its traditional focus on interpersonal dynamics and leader behavior to encompass organizational systems and institutional arrangements that either enable or constrain safe voice. The implication is that psychological safety research and practice must attend to system-level safeguards, not only interpersonal climate.

The findings support and enrich Morrison and Milliken’s (2000) systemic organizational silence construct. Data reveal not merely aggregated individual silence but climate-level collective beliefs that voice generates harm rather than organizational benefit. Vicarious learning from observed punishment of voice is central to this collective belief formation (Bandura, 1977; Newman et al., 2017). This finding supports interventions targeting systemic beliefs and organizational climate, not merely individual voice behavior. It also identifies a cost that is easily overlooked: where employees withhold what they know, the knowledge exchange on which organizational performance partly rests is curtailed at the source (Mohiya, 2026d).

The research extends Edmondson’s (2019) leadership-centered framework by identifying safety-destroying behaviors as a theoretically and practically important complement to safety-building behaviors. Where prior theory has emphasized what leaders do to build safety (invite input, model fallibility, respond non-defensively), this study documents the systematic, destructive effects of threats, public humiliation, and retaliatory conduct — a bidirectional framework with significant practical implications.

Finally, the findings suggest that cultural and structural factors amplify psychological safety barriers through multiplicative rather than merely additive effects. High power distance intensifies fears of challenging authority; collectivist harmony norms compound concerns about relational damage; employment precarity magnifies perceptions of career threat. These interactions demand culturally contextualized theory development (Gelfand et al., 2011).

### 5.3 The Justice–Safety Vicious Cycle: An Integrative Theoretical Contribution

A note on terminology properly precedes the model. Throughout this section, associative language describes conceptual relationships proposed by the integrated theoretical framework and supported thematically by the empirical material; it does not assert statistical association, causal direction, or temporal ordering in the quantitative sense. The Justice–Safety Vicious Cycle Model organizes observed patterns into a coherent explanatory narrative and should be read accordingly.

The study’s most substantive theoretical contribution is the Justice–Safety Vicious Cycle Model, which explains how violations of organizational justice and the degradation of psychological safety interact in self-sustaining, self-amplifying cycles.

The model’s principal theoretical advances are: first, it suggests justice and safety are not merely correlated constructs but mutually constitutive, dynamically interacting phenomena incapable of meaningful independent resolution; second, it specifies a temporal sequence — justice violations → safety erosion → voice suppression → procedural failures → continued injustice → further safety collapse — whose sequencing is critical for intervention design; third, it reveals the self-amplifying momentum of each cycle iteration, explaining why superficial interventions reliably fail; and fourth, it identifies multiple potential intervention leverage points requiring simultaneous activation. Prior research acknowledging justice-safety connections (Carmeli & Gittell, 2009; Simons & Roberson, 2003) has not provided the detailed empirical grounding and formal cycle model this study contributes. Importantly, consistent with the exploratory qualitative methodology employed, these theoretical advances are presented as conceptual propositions warranting future empirical scrutiny rather than as verified causal claims. Cross-validation through quantitative longitudinal studies, as outlined in Section 6.5, is necessary before strong causal conclusions can be drawn.

### 5.4 Novel Empirical Contributions

The research identifies several phenomena that have received limited prior empirical attention. Performance management weaponization — the systematic, deliberate manipulation of performance evaluation processes to punish voice, enforce compliance, and retaliate against complaints — extends research on performance appraisal politics (Cropanzano et al., 2017) by documenting coercive weaponization as a distinct, systematic control mechanism with profound implications for system design.

Employment status intersectional vulnerabilities extend contingent worker research (Liden et al., 2003; Gallagher & McLean Parks, 2001) by demonstrating that contract and agency employees experience not additive but multiplicative risks at the nexus of employment precarity, power asymmetry, and voice constraint — a compounded vulnerability demanding structural rather than individual-level intervention.

The policy-hypocrisy dynamic — in which organizations espousing strong ethical commitments while systematically tolerating violations generate more corrosive cynicism than organizations without such commitments — extends psychological contract breach theory (Morrison & Robinson, 1997; Simons, 2002) by specifying the particularly toxic nature of proclaimed-but-unenforced commitments.

### 5.5 Contextual Dynamics

The research contributes to the field of underrepresented Middle Eastern organizational scholarship (Mellahi & Budhwar, 2010) while illuminating the tension between contextual particularities and universal principles. Context-specific dynamics include high power distance norms that strengthen hierarchical deference expectations, collectivist values that amplify harmony-preservation voice inhibitions, Islamic ethical frameworks that provide powerful normative condemnation of mistreatment, and complex tribal-kinship-national status intersections that create multidimensional power structures. These factors shape the expression, threshold, and response repertoires of mistreatment in organizationally significant ways.

Nevertheless, universal principles emerged unambiguously: fundamental dignity and respect needs proved consistent across all national and cultural backgrounds; justice violations produced negative evaluations regardless of cultural origin; and employees from all backgrounds converged in condemning abusive conduct. The implication is clear: organizations in non-Western contexts cannot invoke cultural relativity to dismiss mistreatment as inappropriate Western impositions. Employees universally value dignity and fairness. Cultural sensitivity in intervention design is essential, but organizational ‘culture’ cannot serve as a legitimizing cover for tolerating abuse.

*Alignment with the Study’s Operational Definition:* It is instructive to assess how the empirical findings align with the operational definition of mistreatment behavior established at the outset of the study. The definition proposed five criteria: (1) violation of dignity and respect norms; (2) cultivation of hostile or demeaning climates; (3) exploitation of asymmetric power; (4) discrimination on categorical characteristics; and (5) suppression of employee voice. All five criteria received robust empirical support across the five themes. Criteria (1) and (2) are most directly evidenced in Theme A; criterion (3) is clarified in both Themes A and C; criterion (4) is documented in Theme C (particularly employment-status discrimination and nepotism); and criterion (5) is the central focus of Theme B. This alignment confirms that the adopted definition was operationally productive and empirically generative, capturing the full range of mistreatment phenomena encountered in the research setting.

*Implications for Western Organizational Contexts*: Although the research is situated in a Saudi Arabian organizational setting, several findings resonate strongly with documented patterns in Western workplaces and carry transferable implications. The dynamics of performance management weaponization, the fear-driven suppression of voice, the policy-practice gap, and the protection of perpetrators at the expense of victims have been documented in Western organizational contexts by Tepper et al. (2017), Morrison (2014), and Cortina and Magley (2009), respectively. These convergences suggest that the Justice–Safety Vicious Cycle Model may have cross-cultural applicability, with cultural context (power distance, collectivism, Islamic values) conditioning the intensity and expression of these dynamics rather than constituting a distinct causal factor. At the same time, the specific mechanisms identified — wasta-mediated favoritism, Vision 2030 generational tensions, and religious-ethical framing of mistreatment — are contextually particular and do not directly transfer to Western settings. The study thus contributes to a cross-cultural organizational behavior literature that emphasizes both universal principles and contextually contingent processes (Gelfand et al., 2011), suggesting that future comparative research should examine how these dynamics manifest across varying cultural configurations.

## 6. Contributions, Practical Implications, Limitations, and Future Directions

### 6.1 Theoretical Contributions Summary

This study makes four substantive theoretical contributions. First, it advances Organizational Justice Theory by demonstrating interactive and compounding justice effects, establishing procedural justice as a psychological safety infrastructure, and extending the theory’s applicability to non-Western hierarchical collectivist contexts. Second, it advances Psychological Safety Theory by identifying safety-destroying leadership behaviors as theoretically important counterparts to safety-building behaviors, extending the theory to encompass organizational systems, and documenting cultural amplification of safety barriers. Third, the Justice–Safety Vicious Cycle Model offers an original integrative theoretical framework formally modeling the bidirectional, self-perpetuating relationship between justice erosion and psychological safety degradation. Fourth, the research identifies novel empirical phenomena — performance management weaponization, intersectional employment-status vulnerabilities, and policy-hypocrisy cynicism — thereby opening productive new research directions.

### 6.2 Practical Recommendations

#### 6.2.1 Establishing Genuine Accountability Architecture

Organizations must implement transparent, procedurally sound mechanisms for misconduct investigations, governed by impartial investigators without conflicts of interest. Behavioral standards must be applied consistently across all organizational levels. Enforcement of stated anti-mistreatment commitments must be visible, consistent, and consequential, rendering ‘zero tolerance’ substantively meaningful. External oversight functions independent of managerial hierarchies offer structural protection against systemic bias.

#### 6.2.2 Constructing Safe and Accessible Voice Infrastructure

Multiple, varied, accessible voice channels — confidential reporting platforms, ombudsperson functions, peer advocate systems — must be established and actively communicated. Meaningful, legally enforceable protections against retaliation for good-faith reporting must be implemented and explicitly publicized. Demonstrated organizational responsiveness to voiced concerns is essential to sustaining belief in the value of voice. Senior leaders should periodically model receptiveness to upward feedback. Channels of this kind further require sustained organizational backing to operate as more than a nominal provision (Mohiya, 2025e).

#### 6.2.3 Dismantling Structural Inequities

Employment status-based differential treatment must be explicitly prohibited and actively monitored. Merit-based, transparent personnel decision processes must displace nepotistic, informal influence systems. Regular organizational justice climate audits across diverse employee segments should inform priorities for structural reform.

#### 6.2.4 Leadership Development and Selection Reform

Supervisory selection criteria must include interpersonal competence, dignity-affirming conduct, and the cultivation of psychological safety as core requirements. Mandatory leadership development programs that emphasize respectful management, non-defensive responses to challenges, justice-informed decision-making, and the routine practice of recognition and thanks should be implemented (Mohiya, 2026c). Behavioral probationary periods for newly appointed supervisors, with structured subordinate feedback mechanisms, should serve as accountability anchors.

#### 6.2.5 Performance Management System Safeguards

Calibration processes involving multiple evaluators and structured appeal mechanisms should be implemented to prevent the weaponization of performance ratings. Formal separation between performance evaluation and misconduct investigation processes is essential. Whistleblower status should be explicitly protected within performance management protocols.

### 6.3 Methodological Contributions

The integration of naturally occurring internal digital discourse data with semi-structured interview data represents a significant methodological contribution. This approach combines the authenticity and lack of social desirability filtering characteristic of naturally occurring data with the depth and probing capacity of interview-based inquiry. The convergence of findings across these epistemologically distinct sources substantially strengthens credibility and extends a triangulation logic applied in earlier qualitative investigations of employee experience (Mohiya, 2024a, 2024b). The study’s approach of systematically applying established theoretical frameworks to qualitative data indicates how theory-driven analysis generates both richness and analytical depth, bridging exploratory qualitative and theory-testing quantitative research traditions.

### 6.4 Limitations

The study’s single-case design limits direct generalizability, though theoretical propositions are offered as transferable insights subject to contextual testing. Self-selection into both data sources may overrepresent employees with heightened salience of mistreatment. Social desirability bias cannot be fully eliminated, even with confidentiality assurances, particularly in high-power-distance contexts. The bounded data collection windows (2014 for the platform corpus; 2017–2020 for the interviews) preclude examination of temporal dynamics. Future research should address these limitations through comparative multi-site designs, longitudinal tracking, and complementary methodologies. A more nuanced self-selection limitation operates in the blog data: not only may employees experiencing mistreatment be overrepresented relative to unaffected employees, but within the group of affected employees, those fearing the most severe retaliation are likely underrepresented, since these individuals are least likely to post on a semi-public platform. This dual selection dynamic means the blog data may simultaneously under-represent both the least affected employees and the most severely silenced victims — leaving a particular stratum (employees who experienced mistreatment but retained some capacity and willingness to voice) as the most visible group. Additionally, the 2014 origin of the blog data introduces a temporal limitation: organizational culture may have evolved in subsequent years. The study’s cross-temporal triangulation with 2017–2020 interview data partially addresses this, though not fully. Furthermore, the social desirability concern applies differentially. While anonymization was applied before analysis, employees posting on a semi-public platform at the time of posting were not anonymous, which likely suppressed the expression of the most sensitive concerns. The interview data, conducted under stronger confidentiality conditions, provide a partial corrective.

### 6.5 Future Research Agenda

The findings suggest six productive research directions: (1) comparative investigation across multiple Saudi organizations varying in sector, ownership, and size; (2) systematic cross-cultural comparison of mistreatment dynamics across Middle Eastern and Western organizational contexts; (3) longitudinal studies tracking the temporal evolution of justice perceptions, psychological safety, and voice behavior; (4) quasi-experimental evaluation of specific interventions — accountability mechanisms, voice infrastructure, leadership development — targeting identified cycle stages; (5) multi-level hierarchical modeling examining nested individual, team, and organizational level dynamics simultaneously, in the spirit of microfoundational accounts that trace organization-level outcomes to individual-level mechanisms (La Sala et al., 2023); and (6) theoretical extension testing boundary conditions of the Justice–Safety Vicious Cycle Model and exploring mechanisms through which virtuous cycles might emerge and sustain.

## Data Availability

The qualitative material underpinning this study comprises anonymized interview transcripts and anonymized comments retrieved from the organization’s internal communication platform. Participants consented to academic use of their accounts under guarantees of anonymity, and verbatim material generated within a single organization carries a residual risk of re-identification; the full corpus is therefore not publicly deposited. The coding framework, the thematic structure, and further anonymized excerpts are available from the corresponding author on reasonable request.

## Appendix A: Semi-Structured Interview Protocol

The following interview guide was used for all thirty-nine semi-structured interviews. Questions were posed in English; where participants preferred Arabic, a bilingual research assistant provided simultaneous facilitation. The semi-structured format allowed probing and follow-up questions based on participants’ responses.

Participant Background (not recorded, for researcher orientation only):

- Years of employment at organization; functional area; approximate level

Section 1: Understanding of Mistreatment Behavior

**1.** In your own words, how would you describe ‘mistreatment’ or ‘unacceptable behavior’ in a workplace context?
**2.** What specific types of behavior do you consider most problematic in this organization?
**3.** How widespread do you believe these behaviors are in your work environment?

Section 2: Personal Experiences and Observations

**4.** Can you describe a situation — either personally experienced or observed — that you would characterize as mistreatment? (Follow-up: What happened? Who was involved? What was the impact?)
**5.** How did this behavior affect you/the person involved — emotionally, professionally, and practically?
**6.** Were there any longer-term consequences that you are aware of?

Section 3: Organizational Responses

**7.** When mistreatment occurs, what typically happens in this organization?
**8.** Have you ever observed a formal complaint or grievance process? What was the outcome?
**9.** How would you describe the organization’s response to reports of mistreatment?

Section 4: Voice and Silence

**10.** Have you personally ever considered raising a concern about mistreatment — formally or informally? What factors influenced your decision?
**11.** Why do you think many employees choose not to report mistreatment, even when they are directly affected?
**12.** What would need to change for you to feel safe raising concerns about mistreatment in this organization?

Section 5: Organizational Systems and Structures

**13.** Are there organizational systems or processes that you believe make mistreatment more likely?
**14.** How does the performance appraisal system relate to mistreatment dynamics, in your observation?
**15.** How do you perceive the difference in treatment between permanent and contract employees?

Section 6: Cultural and Contextual Factors

**16.** In what ways do you think the cultural or social context here shapes how mistreatment occurs or is responded to?
**17.** Are there cultural, religious, or generational factors that, in your view, affect workplace behavior and responses to mistreatment?
**18.** Is there anything else you would like to share about your experiences or observations that we have not covered?
